# Hernia of the Uterus

**Published:** 1849-01

**Authors:** 


### OBSTETRICS.

*Hernia of the Uterus.*—Hysterocele is stated by Mr. Bell, to occur under three forms:—1. Through the inguinal canal. 2. Through the crural ring. 3. Through a separation or rent in the abdominal walls. Two cases of the latter are recorded by him.

1. Mrs. P., delivered of her fifth child eight days previously. When seen she was collapsed, with quick small pulse. She complained of intense pain in the abdomen; on examining which the uterus was found protruding through a rent in the linea alba. It was reduced, but the patient died. No inspection allowed.

2. In a female, confined for the fourth time, after the birth of the child the uterus was felt to have escaped through a rent in the linea alba, and containing another fœtus. The membranes were ruptured after reducing the uterus, and the second child was speedily born. This woman did well.

Cases of inguinal and crural hysterocele are narrated by Sennert, Lallemand, Chopart, and Thurat.

The treatment of ventral hysterocele, consists in reduction, bandaging, and the recumbent posture.-*Prov. Med. Surg. Journ. from Monthly Journal,* July, 1848.

